# Transcript profiling of different types of multiple sclerosis lesions yields FGF1 as a promoter of remyelination

**DOI:** 10.1186/s40478-014-0168-9

**Published:** 2014-12-11

**Authors:** Hema Mohan, Anita Friese, Stefanie Albrecht, Markus Krumbholz, Christina L Elliott, Ariel Arthur, Ramesh Menon, Cinthia Farina, Andreas Junker, Christine Stadelmann, Susan C Barnett, Inge Huitinga, Hartmut Wekerle, Reinhard Hohlfeld, Hans Lassmann, Tanja Kuhlmann, Chris Linington, Edgar Meinl

**Affiliations:** Institute of Clinical Neuroimmunology, Ludwig Maximilian University Munich, Marchioninistraße 15, D-81377 Munich, Germany; Institute of Neuropathology, University Hospital Münster, Münster, Germany; Division of Clinical Neurosciences, University of Glasgow, Glasgow, UK; Division of Neuroscience, Institute of Experimental Neurology (INSpe), San Raffaele Scientific Institute, Milan, Italy; Institute for Neuropathology, Göttingen, Germany; The Netherlands Brain Bank, Netherlands Institute for Neuroscience, Amsterdam, Netherlands; Max Planck Institute of Neurobiology, Hertie Senior Professorship, Martinsried, Germany; Munich Cluster for Systems Neurology (SyNergy), Munich, Germany; Center for Brain Research, Medical University of Vienna, Vienna, Austria; Present address: Department of Neurology, University of Münster, Münster, Germany

**Keywords:** Multiple sclerosis, Remyelination, Demyelination, Fibroblast growth factor

## Abstract

Chronic demyelination is a pathological hallmark of multiple sclerosis (MS). Only a minority of MS lesions remyelinates completely. Enhancing remyelination is, therefore, a major aim of future MS therapies. Here we took a novel approach to identify factors that may inhibit or support endogenous remyelination in MS. We dissected remyelinated, demyelinated active, and demyelinated inactive white matter MS lesions, and compared transcript levels of myelination and inflammation-related genes using quantitative PCR on customized TaqMan Low Density Arrays. In remyelinated lesions, fibroblast growth factor (*FGF) 1* was the most abundant of all analyzed myelination-regulating factors, showed a trend towards higher expression as compared to demyelinated lesions and was significantly higher than in control white matter. Two MS tissue blocks comprised lesions with adjacent de- and remyelinated areas and *FGF1* expression was higher in the remyelinated rim compared to the demyelinated lesion core. In functional experiments, FGF1 accelerated developmental myelination in dissociated mixed cultures and promoted remyelination in slice cultures, whereas it decelerated differentiation of purified primary oligodendrocytes, suggesting that promotion of remyelination by FGF1 is based on an indirect mechanism. The analysis of human astrocyte responses to FGF1 by genome wide expression profiling showed that FGF1 induced the expression of the chemokine *CXCL8* and leukemia inhibitory factor, two factors implicated in recruitment of oligodendrocytes and promotion of remyelination. Together, this study presents a transcript profiling of remyelinated MS lesions and identified FGF1 as a promoter of remyelination. Modulation of FGF family members might improve myelin repair in MS.

**Electronic supplementary material:**

The online version of this article (doi:10.1186/s40478-014-0168-9) contains supplementary material, which is available to authorized users.

## Introduction

The adult central nervous system contains a large pool of mitotic oligodendroglial progenitor cells (OPCs) which rapidly differentiate and remyelinate lesions in models of toxin or immune mediated demyelination [1]. In MS, however, this endogenous repair mechanism frequently fails, resulting in the formation of chronic demyelinated plaques with glial scars, the pathological hallmark of the disease [2,3]. This has profound functional consequences as demyelination not only disrupts saltatory conduction, but also compromises axonal survival by enhancing susceptibility to damage by inflammatory mediators [4], and by disrupting trophic support provided by myelinating oligodendrocytes [5-7]. The frequently observed failure of remyelination in MS does not appear to be due to an intrinsic, absolute defect of myelination, because de- and remyelinated MS lesions are frequently found side by side in the majority of patients. Furthermore, early MS lesions often show signs of remyelination [8-12].

It is unclear why many MS lesions fail to remyelinate. The presence of OPCs and premyelinating oligodendrocytes in many demyelinated lesions [13,14] suggests that this could be caused by failure of OPC to differentiate into myelinating oligodendrocytes. Overcoming this differentiation block to enhance remyelination by endogenous OPC is considered a logical strategy to restore saltatory conduction and reduce accumulation of disability due to axonal pathology [15,16]. Achieving this goal, however, is complicated as a multitude of cellular and molecular changes within the MS lesions can influence OPC migration, survival or differentiation [17-19]. While experimental studies demonstrate that many factors can individually influence OPC migration and/or differentiation [20-32], their relative expression within MS lesions and their significance in modulating remyelination remains unclear. Expression profiling of MS lesions can provide new insight into pathomechanisms [33-35], but this approach has not been applied to remyelinated lesions yet.

To identify endogenous pathways that might be exploited to enhance remyelination we analyzed white matter lesions and used qPCR to focus on the expression of genes regulating oligodendrocytes. We compared dissected remyelinated lesions, actively demyelinating lesions, inactive demyelinated lesions and control white matter. This revealed an important role of the FGF family in the regulation of remyelination. The FGF family is known to regulate oligodendrocyte biology and myelin thickness [36-40], but its role in MS lesion development is unclear. We found that among the analyzed myelination-regulating genes *FGF1* was the most abundant transcript in remyelinated lesions. In two lesions that contained a demyelinated core and a remyelinated rim, the *FGF1* transcript levels were higher in the remyelinated parts suggesting that the increased availability of FGF1 may support remyelination.

FGF1 has been reported to promote proliferation of glial precursors [41], but had so far not been linked to remyelination or its failure in MS. We employed a dissociated myelinating culture system [42-44], a remyelinating slice culture model [45,46], a pure oligodendrocyte culture, and genome wide expression profiling of astrocytes to deduce the functional relevance. This revealed that FGF1 promotes myelination as well as remyelination, presumably via an indirect mechanism. In astrocytes, FGF1 induced leukemia inhibitory factor *(LIF)* and the chemokine *CXCL8*, both implicated in the recruitment of oligodendrocytes and the promotion of remyelination. This suggests that also *in vivo* FGF1 stimulates astrocytes to release factors promoting remyelination and that selective modulation of FGF signaling pathways could provide a novel strategy to enhance remyelination in MS and other demyelinating diseases.

## Materials and methods

### Tissue samples

For mRNA expression analysis we used frozen autoptic tissue samples from the Netherlands Brain Bank, the UK MS Brain Bank, the NeuroResource tissue bank, UCL London, and the Department of Forensic Medicine, LMU Munich. Twelve tissue blocks from 9 MS patients contained 12 white matter lesions, two of them with a demyelinated core and a remyelinated rim. As control, we used 6 control tissue blocks from 4 healthy subjects without clinical or histological evidence of CNS disease (details in Additional file 1: Table S1).

For immunostaining of FGF1 we used 10 formalin fixed paraffin embedded (FFPE) tissue blocks from 7 MS patients containing remyelinated, demyelinated inactive and demyelinated active areas as well as and 3 control tissue blocks from 3 healthy subjects. Most of the patients had secondary progressive MS. The mean disease duration was 24 years, the sex (f/m) ratio was 2:1 (details in Additional file 1: Table S1).

MS lesions were classified according to defined criteria: Active lesions contained abundant macrophages with degraded myelin products visualized by luxol fast blue (LFB) or oil red O staining. Inactive demyelinated lesions were sharply demarcated from the normal appearing white matter and largely devoid of macrophages. Remyelinated lesions were sharply demarcated from the normal appearing white matter and identified by a fainter LFB staining.

Tissue was collected from donors from whom a written informed consent was provided for brain autopsy and the use of the material and clinical information for research purposes.

### Dissection of brain specimens, RNA extraction, cDNA synthesis, and quantitative PCR of brain tissue

Selected areas from tissue blocks were obtained as follows: Cryosections (20 μm) were mounted on PEN slides (P.A.L.M. Microlaser, Bernried, Germany). To identify demyelinated, remyelinated, control white matter, and grey matter areas every 6^th^ section (30 μm) was stained with LFB. The unstained sections were superimposed on LFB stained sections and the lesion areas were marked and macrodissected manually. In total 200–300 μm of each block was used for transcript analysis. To check the precision of dissection, the macrodissected sections were stained with LFB. The control tissue samples used for qPCR exclusively contained white matter. Two MS tissue blocks with adjacent de-and remyelinated lesions were analyzed individually.

RNA was obtained from the dissected tissue specimens by guanidinium thiocyanate-phenol-chloroform extraction (TRI® Reagent, SIGMA, Munich, Germany), and cDNA was synthesized using random hexamers (High Capacity cDNA Reverse Transcription kit, Applied Biosystems (ABI, Darmstadt, Germany). Subsequently, qPCR was performed using custom made TaqMan Low Density Arrays (TLDAs) on the TaqMan 7900 thermocycler (both ABI). These TLDAs are based on qPCR reactions performed in 384 well plates with prespotted primers for the selected genes. We used the qPCR method for our study, because short amplicons (typically < 100 bp) give highly reliable expression data of autoptic tissue, both from frozen and in some cases even from FFPE material [47]. We included genes coding for factors regulating oligodendrocyte development (46), their receptors (20), myelin components (7) and genes that reflect immunological features of the lesions (9). The ciliary neurotrophic factor (CNTF) amplicon we quantified would also detect the ZFP91-CNTF read-through transcript which is thought to be non-coding (http://www.ncbi.nlm.nih.gov/gene/386607). Data were analyzed using RQ Manager 1.2 software (ABI). We applied three housekeeping genes, glyceraldehyde 3-phosphate dehydrogenase (*GAPDH*), β-actin (*ACTB*) and peptidylprolyl isomerase A *(PPIA)*. Differential gene expression was determined in 6 demyelinated inactive lesions from 4 subjects, 4 remyelinated lesions from 3 subjects, 4 demyelinated active lesions from 3 subjects, and 6 control white matter specimens from 4 subjects.

### Histological staining

To characterize the tissue lesions, conventional histochemical analysis like LFB and H&E, as well as immunohistochemistry for CD68 was used. LFB staining was done on 30 μm thick sections for frozen samples and on 4 μm for FFPE samples. Immunostaining for CD68 was performed with mouse peroxidase anti peroxidase (PAP) (Dako, Eching, Germany) system using diaminobenzidine (DAB) (DAKO) as the chromogenic substrate.

For FGF1 single staining with DAB, as well as the immunofluorescence staining, additional signal amplification was required. Tyramide signal amplification Plus Biotin kit (PerkinElmer, Waltham, USA) was used according to manufacturer instructions. To perform double staining, immunofluorescence was performed on FFPE sections. The sections (4 μm) were first deparaffinised and antigen retrieval was performed by boiling the slides for 30 min in Tris-EDTA buffer. To detect FGF1 we applied the mAb 2E12 (ab117640, Abcam, Cambridge, UK). This mAb recognizes FGF1 by Western blot, immunocytochemistry and flow cytometry, but we cannot exclude some crossreactivity to other FGF family members, since the epitope of this mAb has not yet been mapped (information from Abcam). Other primary antibodies used were specific for GFAP (Dako), Iba-1 (Wako, Neuss, Germany), CD20 (Epitomics, Burlingame, USA) and CD3 (AbD Serotec, Kidlington, UK). Sections were incubated with the primary antibody either for 2 h at RT or overnight at 4°C. The following secondary antibodies were used: goat anti-mouse IgG3 Alexa 488, goat anti-mouse IgG2a Alexa 488, goat anti-rabbit IgG Alexa 488 (Invitrogen, Karlsruhe, Germany), rat anti-mouse IgG1 biotinylated (BD, Heidelberg, Germany) and streptavidin Alexa 568 (Invitrogen). Negative controls included either isotype specific antibody or the purified IgG.

All images were acquired on either Leica LMD7000 or Leica DMI6000 microscopes (Leica Microsystems, Wetzlar, Germany) and processed using ImageJ software (NIH).

### *In vitro* myelination (dissociated spinal cord cultures)

*In vitro* myelinating cultures were prepared as described before [42-44]. Briefly, neurosphere derived astrocytes were grown to confluence. Spinal cord cell suspensions from either E15.5 Wistar or Sprague Dawley outbred rat strains were plated on the neurosphere derived astrocytes. The plating media contained 50% DMEM, 25% heat inactivated horse serum, 25% HBSS with Ca^2+^ and Mg^2+^, and 2 mM L-glutamine. Spinal cord cells were plated in a density of 150,000 cells on a 13 mm cover slip, coated with poly-L-lysine. Cells were allowed to attach for 2 h at 37°C and subsequently, the plating medium was filled up with differentiation medium, containing DMEM (4.5 mg/ml glucose), 10 ng/ml biotin (Sigma), 0.5% N1 hormone mixture, 50 nM hydrocortisone, and 0.5 mg/ml insulin (Sigma). If not mentioned specifically, all the reagents used were from Invitrogen. The final ratio of plating- and differentiation-medium was 50/50. Three 13 mm coverslips were kept in 3.5 cm petri dishes with a total medium volume of 1 ml. Cultures were maintained at 37°C in 7% CO_2_ and fed three times a week by replacing half the culture medium with fresh differentiation media. After twelve days insulin was omitted from the culture medium. The cultures were maintained for 26–28 days (as indicated in the experiment). For stimulation, recombinant human FGF1 was added together with fresh differentiation medium.

To access myelination immunocytochemistry was used. Briefly, cells were fixed with 4% PFA for 20 min at RT, washed in PBS and permeabilized with 0.5% Triton X-100 and 0.5% porcine gelatin for 20 min at RT. Primary antibodies used for the staining were specific for neurofilament (NFL), (SMI-31; Abcam), myelin basic protein (MBP) (Millipore) or myelin oligodendrocyte glycoprotein (MOG). Secondary antibodies used were: Goat anti-mouse IgG2a Alexa 488 and goat anti-mouse IgG1 Alexa 568 (Molecular Probes). Primary antibodies were incubated overnight at 4°C. Secondary, fluorescence labeled antibodies were incubated for 45 min at room temperature and cover slips were mounted with Vectashield (Vector laboratories, Peterborough, UK).

For imaging and quantitative analysis 20–30 images per treatment per experiment were acquired at random with a 10X objective using ZEISS AxioVert200M or Olympus BX51 fluorescent microscope. For each condition 3 coverslips were quantified in each experiment. MBP and SMI-31 staining were measured as the pixel value related to the total pixel number in the image. The pixel values were calculated using either Metamorph software (series 7.7) or Image J (NIH systems, version 1.45) combined with Adobe Photoshop (Elements 7.0). To determine the percentage of myelination in each coverslip the number of MBP positive pixels was divided by the number of SMI-31 positive pixels. Significance of data values was analyzed using T-test.

### Organotypic cerebellar slice cultures

Organotypic cerebellar slice cultures were prepared and cultured as previously described [48]. Briefly, cerebellum and attached hindbrain were extracted from newborn (P0) CD1 mouse pups (Charles River Laboratories) and cut into 300 μm sagittal sections using a McIlwain tissue chopper. Slices were separated and transferred on Millicell-CM culture inserts (Millipore, Darmstadt, Germany) in 6-well plates. Culture media was composed of 50% minimal essential media, 25% heat-inactivated horse serum, 25% Earle’s balanced salt solution, 6.5 mg/ml glucose, and penicillin-streptomycin and Glutamax. Membranes were transferred into fresh media every 2–3 days. Slices were left untreated for 12 days *in vitro* to allow to myelinate [45]. Subsequently, slices were demyelinated with lysolecithin (0.5 mg/ml, 16 h), washed in PBS and allowed to recover in culture media for 1 day. Afterwards, the slices were treated with FGF1 (100 ng/ml) for 7 days and 14 days, respectively. FGF1 was diluted in culture media and replaced every 2–3 days. Control slices remained untreated during the remyelination phase. Although these cultures are used to study remyelination, we are aware that they may still be equivalent of developmental myelination as the cultures were still at an *ex vivo* stage during which myelination is continuing in the *in vivo* cerebellum.

Total RNA from cultured cerebellar slices were isolated using the RNeasy Micro Kit (Qiagen, Hilden, Germany) according to manufacturer’s protocol. cDNA was generated using the High Capacity cDNA Transcription Kit (ABI). All qPCR analysis was conducted using the StepOne Plus real time cycler (ABI) and the Power SYBR Green Master Mix (ABI). All qPCR results were normalized to human acid ribosomal protein (*hARP*).

For immunostaining, slices were fixed in 4% PFA for 1 hour, and then washed twice in PBS. Subsequently the slices were blocked for 3 h at room temperature. The primary antibody used recognized MBP (DAKO) and NFL (DAKO). As secondary antibodies we used goat anti-rabbit Cy3 and goat anti-mouse Cy2 (Dianova, Germany). Primary antibody was incubated for 48 h at 4°C, and the secondary, fluorescence labeled antibodies were incubated overnight at 4°C and the slices were mounted using Fluorescence Mounting Medium (DAKO).

From these, representative maximal-projection images were generated. The area ratio of MBP to NFL immunostaining was determined by drawing along only the filamentous structures per image and calculated the area using ImageJ. Per condition three animals were used for each of the three experiments.

### Preparation of primary oligodendrocyte progenitor cells (OPCs)

Primary OPCs were isolated using the immunopanning method as described earlier [49]. Briefly, the dissected and chopped forebrains from P6 – P9 day old C57Bl/6 mice were incubated for 20 min at 37°C and 5% CO_2_ in papain buffer, titruated in ovomucoid solution (Cell Systems GmbH, Troisdorf, Germany) and centrifuged at 1000 rpm for 10 min. The cell pellet was resuspended in panning buffer and transferred to a negative selection plate coated with Anti-BSL 1 Griffonia simplificonia lectin (L-1100, Vector Labs/Biozol, Eching, Germany). The supernatant was transferred to a positive selection plate coated with rat anti-mouse CD140a (10R-CD140AMS, Research Diagnostics/Bioleague, Poggensee, Germany) as primary antibody and AffiniPure goat anti-rat IgG (H + L) (112-005-003, Dianova) as secondary antibody. After incubation the adherent OPCs were detached by using a cell scraper, centrifuged for 10 min at 1000 rpm, resuspended in mouse OPC Sato media [49] and plated in T75 culture flask coated with poly-L-lysine. The OPCs were cultured at 37°C and 5% CO_2_; PDGF-AA (10 ng/ml, Peprotech) was added every day. Half of the media was changed every second or third day. The purity of the OPCs cultures was more than 95%.

### Assessment of oligodendroglial proliferation and differentiation

Proliferation of OPCs was determined using BrdU incorporation (Cell Proliferation ELISA, BrdU [colorimetric], Roche Diagnostics, Penzberg, Germany). Oligodendroglial differentiation was assessed by morphology. To allow differentiation of OPCs into oligodendrocytes, PDGF-AA was replaced by 10 ng/ml CNTF (Peprotech). Brightfield pictures were taken after 48 h. At least 100 cells per time point were classified as oligodendroglial progenitor (0–2 processes), immature (3–13 processes) or mature (differentiated cells with myelin sheet formation) oligodendrocytes.

### RNA isolation and qRT-PCR from oligodendrocyte and slice cultures

Total RNA was isolated using peqGOLD Total RNA Kit and mRNA was transcribed into cDNA by reverse transcription reaction (High Capacity cDNA Transcription Kit, ABI). qRT-PCR was performed using Power SYBR® Green PCR Master Mix (ABI) and StepOne Plus real time cycler (ABI). The following primers were used: hARP forward 5’ CGACCTGGAAGTCCA-ACTAC 3’; hARP reverse 5’ ATCTGCTGCATCTGCTTG 3’; MBP for 5’ GTACAAG-GACTCACACACGAGA 3’; MBP rev 5’ GTTCGAGGTGTCACAATGTTCT 3’; MAG for 5’ ACCGCCTTCAACCTGTCTGT 3’; MAG rev 5’ CTCGTTCACAGTCACGTTGC 3’; MOG for 5’ CCTGCAGCACAGACTGAGAGGAAAA 3’; MOG rev 5’ TGCTGGGC-TCTCCTTCCGCT 3’.

### Primary astrocyte cell culture, genome wide transcriptome analysis and RT-qPCR analysis

Human astrocytes of embryonic origin [50] were cultured as described [51]. Medium was changed to serum-free Panserin 401 (PAN Biotech, Aidenbach, Germany) supplemented with 1% penicillin-streptomycin 24 h prior to stimulation with recombinant human FGF1 (10 ng/ml; R&D Systems) and 5 U/ml Heparin-Natrium-25000-ratiopharm® (ratiopharm, Ulm, Germany). Astrocytes were stimulated for 8 and 24 h with FGF1 + heparin or heparin alone. Triplicates were analysed for each time point. Total RNA was extracted using RNeasy Mini Kit (Qiagen). 200 ng of total RNA was spiked with polyadenylated transcripts using the Gene Chip® Poly-A Control kit (Affymetrix, Santa Clara, USA). cRNA was generated and transcribed into anti-strand and strand cDNA. The strand cDNA was purified using the Ambion WT Expression kit (Life Technologies, Carlsbad, USA) and labeled with the Affymetrix GeneChip® WT Terminal Labeling and Control kit (Affymetrix, Santa Clara, USA).

Samples were hybridized to Affymetrix GeneChip® Human Gene 1.0 ST Arrays, containing about 29000 probesets representing annotated human transcripts present in NCBI-RefSeq database. The arrays were scanned using the GeneChip® 3000 scanner and the transcriptome data were exported using AGCC Scan Control software v3.2.3.1515. Subsequently data processing and analysis were performed in R/Bioconductor platform (http://www.bioconductor.org). The data were subjected to robust multi-array (RMA) normalization [52]. Further, 26839 probesets passed detection above background (DABG) p-value < 0.05 in at least 25% of the samples and were considered for the subsequent analysis. No outlier samples were identified by principal component analysis and unsupervised hierarchical clustering based on filtered genes. Differential expression analysis was performed using ebayes test followed by Benjamini & Hochberg method for multiple testing correction, as implemented in the LIMMA bioconductor package [53]. Differentially expressed probes were defined by three criteria: (a) corrected p-value <0.05, (b) fold-change threshold ± 1.4 (c), normalized mean expression intensity > = 100 in any one of the two groups.

qPCR was performed using TaqMan® assays (ABI) to quantify the expression of *HMOX1* (Hs01110250_m1), *CXCL8* (Hs00174103_m1), *LIF* (Hs00171455_m1), and *PPIA* (4326316E). TaqMan® PCR Core Reagent Kit (ABI) was applied, PCR was performed on the 7900HT Fast Real-Time PCR System (ABI) and SDS software version 2.3 (ABI) was used for data analysis.

### ELISA

We used ELISA assays to detect human CXCL8 (#DY208) and human LIF (#DY7734-05) (R&D Systems). 3.3′,5.5′-Tetramethylbenzidine (TMB) solution (Sigma-Aldrich) was used as substrate and color reaction was stopped by adding 1 mol/l H_2_SO_4_. Absorption was measured at 450 nm and 540 nm.

## Results

### Astrocyte activation and immune cells in remyelinated lesions

*CD68* and *HLA-DR* expression was highest in the active lesions (Table 1). Transcripts for myelin associated genes were greatly reduced in the demyelinated inactive lesions compared to remyelinated lesions (Table 1). The myelin associated genes, were however, not completely absent in demyelinated lesions (Table 1), an observation which can be explained by the presence of premyelinating oligodendrocytes in the demyelinated lesions [13]. A heat map of all analyzed de- and remyelinated lesions, which were dissected based on LFB staining, shows that our qPCR results reflected the established pathology of MS lesions (Additional file 2: Figure S1).

**Table 1:** **Expression levels of myelin genes, astrocyte and inflammation markers in MS lesions and their fold-changes in demyelinated inactive versus remyelinated lesions**

**Gene name**	**CWM**		**Remyelinated**		**Demyelinated**		**Active**		***Re/CWM***		***Re/De***	
	**Mean**	**SEM**	**Mean**	**SEM**	**Mean**	**SEM**	**Mean**	**SEM**	***Ratio***	**p**	***Ratio***	**p**
**Myelin proteins**												
*CNP*	**333.38**	75.99	**539.31**	253.33	**50.77**	20.00	**228.49**	77.20	*1.62*	0.48	*10.62*	***0.0095***
*MAG*	**57.21**	10.00	**65.82**	11.80	**11.57**	6.28	**43.18**	4.67	*1.15*	0.76	*5.69*	***0.019***
*MBP*	**40.84**	7.40	**41.85**	13.02	**6.91**	2.49	**107.43**	83.98	*1.02*	0.76	*6.06*	***0.0095***
*MOBP*	**350.12**	84.65	**422.56**	164.85	**34.10**	18.05	**316.04**	145.56	*1.21*	0.91	*12.39*	***0.0095***
*MOG*	**61.33**	15.60	**90.21**	13.05	**10.87**	5.26	**50.26**	14.17	*1.47*	0.26	*8.30*	***0.0095***
*OMG*	**48.67**	13.67	**86.83**	32.00	**13.87**	4.27	**20.85**	4.88	*1.78*	0.35	*6.26*	***0.019***
*PLP1*	**2239.24**	486.85	**3434.69**	397.98.	**400.80**	143.30	**1917.75**	252.49	*1.53*	0.11	*8.57*	***0.0095***
**Astrocyte and inflammation markers**												
*GFAP*	**890.74**	123.37	**2713.08**	451.88	**4715.80**	1637.94	**11466.90**	9726.52	*3.05*	**0.010**	*0.58*	*0.91*
*CD68*	**5.57**	0.68	**11.24**	1.00	**12.07**	3.67	**62.60**	33.16	*2.02*	**0.010**	*0.93*	*1*
*HLA-DRA*	**21.83**	6.34	**29.63**	8.83	**20.66**	4.05	**79.79**	7.09	*1.36*	0.48	*1.43*	*0.35*
*IL1B*	**0.17**	0.02	**0.14**	0.06	**0.19**	0.10	**0.33**	0.12	*0.80*	0.76	*0.71*	*1*
*IL10*	**0.13**	0.03	**0.24**	0.06	**0.07**	0.03	**0.71**	0.40	*1.82*	0.26	*3.29*	***0.040***
*TNFSF13B (BAFF)*	**0.71**	0.17	**2.04**	0.64	**0.95**	0.27	**2.88**	1.10	*2.88*	0.067	*2.15*	*0.26*
*TGFB1*	**5.32**	0.99	**12.68**	2.10	**15.03**	6.22	**106.86**	97.86	*2.38*	**0.010**	*0.84*	*1*
*TGFB2*	**2.02**	0.16	**5.08**	0.86	**7.35**	2.66	**5.36**	0.63	*2.52*	**0.010**	*0.69*	*0.76*
*TGFB3*	**4.73**	0.70	**9.75**	4.75	**3.09**	0.77	**8.10**	5.37	*2.06*	0.35	*3.15*	*0.067*

CWM: Control white matter, Remyelinated: Remyelinated lesion, Demyelinated: Demyelinated inactive lesion, Active: Demyelinated active lesion, Re/CWM and De/Re ratio: Remyelinated vs. control white matter and vs. demyelinated inactive lesions, respectively; p value were calculated by 2-sided U tests, unadjusted for multiple testing. p-values < 0.05 are printed in bold. Specimens analyzed: Six normal white matter specimens from 4 subjects, 6 demyelinated inactive lesions from 4 subjects, 4 demyelinated active lesions from 3 subjects, and 4 remyelinated lesions from 3 subjects were dissected and used for qPCR analysis. The mean expression values are given as % *GAPDH*; SEM denotes standard error of the mean.

In remyelinated lesions, glial fibrillary acidic protein (*GFAP*) was significantly higher expressed than in control white matter, indicating a persistent astrocytic activation in the presence of repaired myelin (Table 1). *CD68,* a marker for microglia activation and macrophage infiltration was also significantly elevated in remyelinated lesions (Table 1). B cell activating factor of the TNF family (*BAFF*), a B cell survival factor was also high in remyelinated lesions (Table 1), which might reflect the persisting astrocytic activation [51].

### Altered expression of oligodendrocyte regulators in de- and remyelinated MS lesions

We quantified the expression of mediators regulating oligodendrocytes focusing on semaphorins, FGFs, PDGFs, chemokines, IL-6 and IGF family members (Table 2) as well as their receptors in the three different lesion types and control white matter (Additional file 3: Table S2).

**Table 2:** **Expression levels of genes potentially regulating oligodendrocytes and their fold-changes in demyelinated inactive versus remyelinated lesions**

**Gene name**	**CWM**		**Remyelinated**		**Demyelinated**		**Active**		***Re/CWM***		***Re/De***	
	**Mean**	**SEM**	**Mean**	**SEM**	**Mean**	**SEM**	**Mean**	**SEM**	***Ratio***	**p**	***Ratio***	**p**
**PDGFs**												
*PDGFA*	**19.38**	2.59	**31.78**	10.91	**15.45**	4.47	**51.82**	34.22	*1.64*	0.61	*2.06*	*0.26*
*PDGFB*	**1.23**	0.28	**1.71**	0.30	**6.79**	2.91	**31.65**	29.42	*1.39*	0.26	*0.25*	*0.48*
*PDGFC*	**0.52**	0.09	**1.45**	0.47	**1.19**	0.48	**2.56**	1.69	*2.78*	**0.038**	*1.22*	*0.61*
*PDGFD*	**0.14**	0.05	**0.37**	0.22	**0.83**	0.46	**0.08**	0.03	*2.61*	1.00	*0.45*	*0.45*
**FGFs**												
*FGF1*	**39.42**	2.61	**85.40**	11.54	**48.15**	19.53	**58.18**	16.80	*2.17*	**0.010**	*1.77*	*0.11*
*FGF2*	**3.82**	0.56	**9.23**	0.88	**10.25**	2.62	**6.08**	0.63	*2.41*	**0.010**	*0.90*	*0.91*
*FGF5*	**0.01**	0.01	**0.00**	0.00	**0.01**	0.01	**0.00**	0.00	0.00*	0.15	0.00*	*0.15*
*FGF8*	**0.00**	0.00	**0.06**	0.05	**0.02**	0.01	**0.01**	0.00	16.25*	0.74	2.69*	*0.72*
*FGF9*	**0.04**	0.02	**0.02**	0.01	**0.87**	0.53	**0.01**	0.00	0.44*	0.71	0.02*	*0.066*
**Semaphorins**												
*SEMA3A*	**0.58**	0.11	**0.77**	0.10	**1.77**	0.75	**0.71**	0.25	*1.32*	0.26	*0.43*	*0.35*
*SEMA3B*	**6.33**	1.73	**10.48**	4.21	**2.82**	1.50	**5.37**	3.57	*1.66*	0.61	*3.72*	*0.11*
*SEMA3C*	**4.84**	1.06	**7.13**	0.75	**2.10**	0.59	**6.13**	1.73	*1.47*	0.17	*3.40*	***0.010***
*SEMA3D*	**0.55**	0.09	**2.18**	0.57	**1.08**	0.37	**0.85**	0.23	*3.94*	**0.019**	*2.02*	*0.11*
*SEMA3E*	**0.41**	0.04	**0.91**	0.29	**0.49**	0.15	**0.69**	0.23	*2.21*	0.26	*1.84*	*0.17*
*SEMA3F*	**0.02**	0.01	**0.02**	0.01	**0.20**	0.10	**1.91**	1.89	0.93*	0.91	0.10*	*0.44*
*SEMA3G*	**0.23**	0.05	**0.16**	0.06	**0.54**	0.31	**1.18**	0.98	*0.68*	0.48	*0.30*	*0.91*
*SEMA4A*	**0.22**	0.06	**0.21**	0.05	**1.00**	0.36	**0.29**	0.04	*0.95*	0.91	*0.21*	*0.067*
*SEMA4B*	**2.63**	0.31	**8.90**	2.16	**8.46**	3.46	**18.31**	14.74	*3.39*	**0.010**	*1.05*	*0.48*
*SEMA4C*	**4.49**	0.77	**8.07**	2.81	**3.67**	0.83	**9.90**	6.17	*1.80*	0.26	*2.20*	*0.11*
*SEMA4D*	**16.61**	3.51	**24.36**	7.25	**4.72**	2.38	**23.80**	11.36	*1.47*	0.61	*5.16*	***0.019***
*SEMA4F*	**0.40**	0.04	**1.27**	0.13	**3.09**	0.67	**4.83**	4.19	*3.18*	**0.010**	*0.41*	*0.11*
*SEMA4G*	**0.35**	0.07	**0.44**	0.13	**0.48**	0.28	**0.89**	0.43	*1.26*	0.61	*0.91*	*0.59*
*SEMA5A*	**2.38**	0.56	**3.06**	0.45	**3.08**	1.07	**8.98**	7.60	*1.28*	0.61	*0.99*	*0.91*
*SEMA5B*	**0.45**	0.09	**0.78**	0.18	**2.34**	0.52	**1.32**	0.53	*1.72*	0.17	*0.33*	*0.11*
*SEMA6A*	**28.24**	6.78	**45.65**	12.14	**12.75**	3.45	**31.24**	4.13	*1.62*	0.26	*3.58*	***0.010***
*SEMA6B*	**0.73**	0.18	**1.95**	0.78	**9.90**	4.74	**31.22**	30.80	*2.67*	0.07	*0.20*	*0.17*
*SEMA6C*	**0.05**	0.01	**0.07**	0.03	**0.13**	0.06	**0.06**	0.02	*1.53*	0.28	*0.54*	*0.83*
*SEMA6D*	**7.30**	1.55	**13.26**	2.93	**4.43**	0.84	**5.70**	1.43	*1.82*	0.11	*3.00*	***0.019***
*SEMA7A*	**2.85**	0.75	**3.91**	0.35	**0.93**	0.50	**2.99**	1.56	*1.37*	0.48	*4.22*	***0.019***
**Chemokines**												
*CXCL1*	**0.18**	0.07	**1.18**	0.32	**4.46**	3.25	**1.30**	1.04	*6.51*	**0.019**	*0.27*	*0.76*
*CXCL2*	**0.17**	0.08	**0.62**	0.14	**1.10**	0.47	**3.49**	2.96	*3.70*	**0.019**	*0.56*	*0.91*
*CXCL8*	**0.23**	0.06	**0.51**	0.19	**0.71**	0.15	**0.52**	0.42	*2.22*	0.26	*0.71*	*0.61*
*CXCL10*	**0.02**	0.01	**0.05**	0.02	**0.04**	0.03	**0.88**	0.47	1.94*	0.76	*1.09*	*0.66*
*CXCL12*	**2.51**	0.37	**3.65**	0.70	**30.09**	23.06	**24.58**	16.09	*1.45*	0.35	*0.12*	*0.76*
**IL6 family**												
*CNTF*	**0.19**	0.04	**0.52**	0.11	**0.20**	0.11	**0.69**	0.50	*2.66*	**0.019**	2.65*	*0.069*
*CLCF1*	**0.02**	0.01	**0.16**	0.13	**0.48**	0.23	**0.55**	0.34	6.62*	0.35	*0.34*	*0.83*
*CTF1*	**0.17**	0.03	**0.44**	0.13	**0.82**	0.29	**1.66**	1.36	*2.59*	0.17	*0.54*	*0.61*
*IL6*	**0.06**	0.02	**0.07**	0.03	**0.38**	0.31	**0.06**	0.02	*1.27*	0.76	*0.19*	*0.83*
*IL11*	**0.01**	0.00	**0.07**	0.05	**0.04**	0.03	**0.02**	0.01	5.11*	1.00	*1.63*	*0.91*
*LIF*	**0.01**	0.00	**0.03**	0.02	**0.76**	0.70	**0.43**	0.37	5.17*	0.40	*0.04*	*1*
**IGFs**												
*IGF1*	**0.35**	0.10	**0.32**	0.12	**0.63**	0.23	**1.46**	0.98	*0.94*	1.00	*0.51*	*0.61*
*IGF2*	**0.23**	0.01	**0.33**	0.12	**6.04**	4.62	**1.95**	1.23	*1.42*	0.26	*0.06*	*0.39*
**Miscellaneous**												
*CTGF*	**1.70**	0.50	**4.53**	0.90	**13.40**	4.85	**9.77**	7.80	*2.67*	**0.038**	*0.34*	*0.26*
*FIGF*	**0.25**	0.07	**0.28**	0.07	**0.60**	0.51	**0.29**	0.10	*1.09*	0.76	*0.46*	*0.34*
*HGF*	**0.12**	0.02	**0.37**	0.12	**0.31**	0.14	**0.99**	0.40	*3.05*	0.26	*1.17*	*0.45*
*LINGO1*	**0.15**	0.11	**0.77**	0.30	**1.42**	0.50	**0.40**	0.14	*5.18*	0.06	*0.54*	*0.61*

CWM: Control white matter, Remyelinated: Remyelinated lesion, Demyelinated: Demyelinated inactive lesion, Active: Demyelinated active lesion, Re/CWM and De/Re ratio: remyelinated vs. control white matter and vs. demyelinated inactive lesions, respectively; p value were calculated by 2-sided U tests, unadjusted for multiple testing. p-values < 0.05 are printed in bold. 0.00: Values were below our detection limit, which was 0.01% *GAPDH.* Specimens analyzed: 6 normal white matter specimens from 4 subjects, 6 demyelinated inactive lesions from 4 subjects, 4 demyelinated active lesions from 3 subjects, and 4 remyelinated lesions from 3 subjects were dissected and used for qPCR analysis. The mean expression values are given as % *GAPDH*; SEM denotes standard error of the mean. *Ratios may be imprecise since at least one value was below or close to the detection limit.

*SEMA3C, SEMA4D, SEMA6A, SEMA6D,* and *SEMA7A* were significantly higher expressed in remyelinated lesions as compared to demyelinated lesions. In contrast *SEMA4A* showed a trend towards higher expression in demyelinated lesions (4.7 fold; p = 0.067; Table 2).

*LINGO-1* (official gene name *LRRN6A*) is of great current interest, because it is tested as a therapeutic target for MS (EUCTR2011-006262-40-CZ) or First Episode of Acute Optic Neuritis (EUCTR2011-006291-39-SE) [54]. Therefore we provide detailed expression data for this gene in the different types of MS lesions and control tissue although its biology was not further studied here (Additional file 4: Figure S2).

The receptors *ERBB3, FGFR2* and *MET* were significantly higher expressed in re- versus demyelinated lesions (Additional file 3: Table S2). *FGFR1, FGFR2, PDGFRB, IL6ST, PLXNB1* and *CXCR7* were significantly higher expressed in remyelinated lesions compared to control white matter (Additional file 3: Table S2).

### *FGF1* is abundant in white matter and further increased in remyelinated lesions

**Figure 1:**
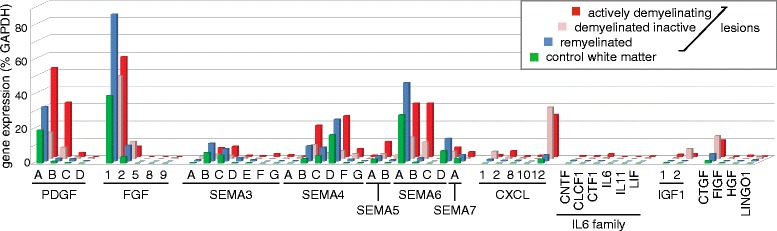
**Oligodendrocyte regulators are differentially expressed in various lesion types.** MS lesions were dissected from frozen tissue and the expression level of the indicated mediators regulating oligodendrocytes were determined by qPCR. The absolute expression levels are given in terms of *% GAPDH*. Displayed is the mean of 6 normal white matter specimens, 6 demyelinated inactive, 4 demyelinated active, and 4 remyelinated lesion areas.

**Figure 2:**
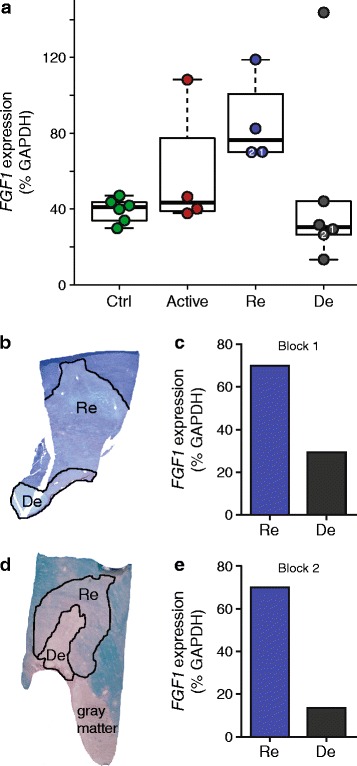
**FGF1 expression is elevated in remyelinated lesions. (a)** *FGF1* gene expression was analyzed by quantitative PCR TLDA relative to *GAPDH* in individual lesion areas (Re: remyelinated, De: demyelinated inactive, Active: actively demyelinating areas) and control white matter specimens (Ctrl.)*.* Each symbol represents a single dissected area. Medians (bars) and 1^st^/3^rd^ quartiles (boxes) are shown. Whiskers extend to the range up to 1.5 times of the interquartile range; values beyond were regarded as outliers. We noted one outlier in the six analyzed demyelinated inactive lesions, but we cannot explain why this one lesion had a higher *FGF1* level than all others. Regarding the primary topic of remyelination in this study, we compared the remyelinated lesions with the other groups of tissue specimens; Mann–Whitney U test showed a significant difference between the remyelinated areas and controls (p < 0.01), while the differences between remyelinated vs. demyelinated inactive areas (p = 0.1) and remyelinated vs. actively demyelinating areas (p = 0.2) did not reach statistical significance. **(b-d)** In two blocks, adjacent de- and remyelinated areas were present within the same lesion and excised for quantitative PCR TLDA analysis (labelled as Block 1 and 2 for Figure 2a-e). *FGF1* expression normalized to *GAPDH* is shown. Fold-changes of *FGF1* expression between the re- and demyelinated areas in each block were calculated for the different housekeeping genes. The geometric mean of these fold-changes obtained by normalization to the three housekeeping genes was 2.1× for block 1 and 4.1× for block 2.

*FGF1* was the most abundant FGF both in the white matter of the control brain and in the white matter of MS lesions (Figure 1, Table 2). Further, *FGF1* had the highest transcript level of all the analyzed myelination-related factors (Figure 1, Table 2) and was therefore further studied in detail. In remyelinated lesions, *FGF1* showed a trend towards higher expression compared to demyelinated lesions and was significantly higher expressed than in control white matter (Figure 1, Figure 2a,b). We could directly compare *FGF1* expression in two tissue blocks with de- and remyelinated areas within the same lesion: in both blocks *FGF1* transcript levels were higher in the dissected remyelinated areas compared to the demyelinated lesion core (Figure 2b-e).

**Figure 3:**
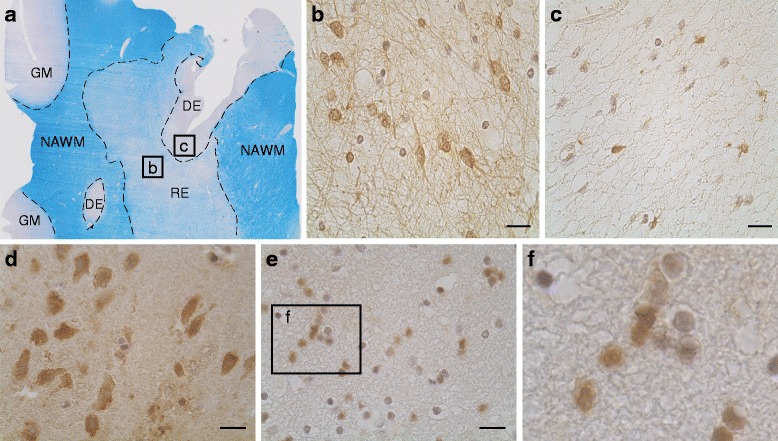
**FGF1 is expressed in re- and demyelinated lesions as well as in neurons and oligodendroglia in control brain and NAWM. (a)** LFB staining of an MS tissue specimen with demyelinated (De), remyelinated (Re) lesion areas along with normal appearing white mater (NAWM) and gray matter (GM). The FGF staining was more prominent in the remyelinated area **(b)** compared to the chronic inactive demyelinated area **(c)**. In control brain, FGF1 staining was detected in neurons of gray matter **(d)** and cells appearing as oligodendrocytes in white matter **(e, f)**. Scale bars: 20 μm.

**Figure 4:**
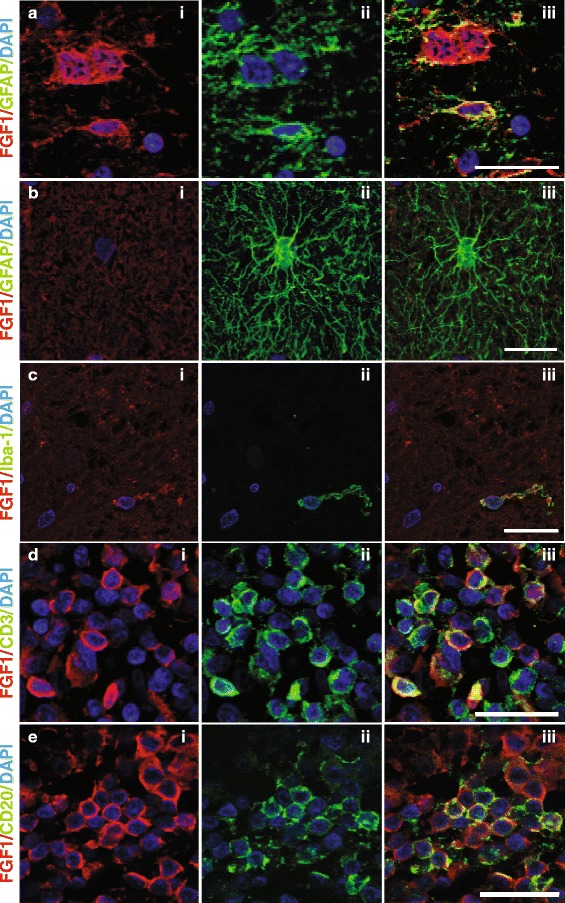
**FGF1 is displayed by astrocytes, microglia/macrophages and lymphocytes.** Double immunofluorescence staining of FGF1 with GFAP **(a, b)**, Iba-1 **(c)**, CD3 **(d)**, and CD20 **(e)**. Pictures were taken from different areas, namely a chronic inactive demyelinated lesion **(a)**, NAWM **(b)**, chronic active lesion **(c)**, and perivascular cuffs in an active lesion **(d, e)**. Scale bars: a and b 12.5 μm; **(c, d)** and **(e)** 25 μm.

Accordingly, FGF1 staining was more prominent in the re- compared to the demyelinated lesion area (Figure 3a-c). Analyzing control brain for FGF1 expression, we found cortical neurons to be FGF1 positive (Figure 3d). Furthermore, FGF1 staining was detected in cells which, according to their morphology, could be oligodendroglia (Figure 3e-f). These FGF1-positive oligodendroglia could also be detected in NAWM of MS brain (data not shown). Double staining of FGF1 and GFAP revealed astrocytes in remyelinated lesions to be FGF1 positive (Figure 4a). In contrast, reactive astrocytes detected in NAWM surrounding the lesion areas were FGF1 negative (Figure 4b). In active lesions we detected subsets of microglia/macrophages to be FGF1 positive (Figure 4c). Furthermore, subsets of B and T cells present in perivascular cuffs displayed FGF1 staining (Figure 4d, e). Together, our staining localized FGF1 to astrocytes, oligodendrocytes, microglia/macrophages and infiltrating lymphocytes, but we cannot conclude from our staining which cells are the major sources and why it is produced.

### FGF1 accelerates developmental myelination in dissociated cultures

*FGF1* was the most abundant of all the myelination-related mediators included in this study (Table 2) and was further upregulated in remyelinated lesions. This led us to speculate that increased availability of this member of the FGF family supported lesion repair. The validity of this concept was first explored in myelinating cultures derived from embryonic rat spinal cord in which myelination is initiated after 12 days *in vitro* and reaches a plateau two weeks later [42,44].

**Figure 5:**
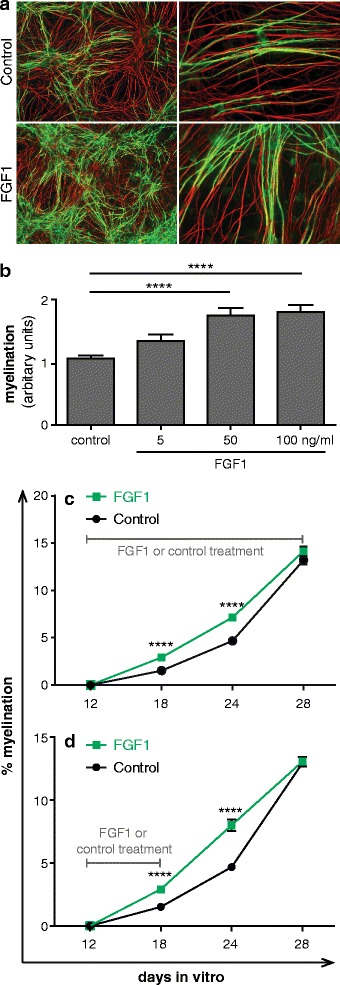
**FGF1 promotes myelination in dissociated spinal cord cultures.** Myelinating cultures were treated with different concentrations of FGF1 from 22 DIV to 26 DIV and then stained for myelin (MBP in green) and axons (SMI-31 in red). **(a)** FGF1 treated cultures showed enhanced levels of MBP^+^ myelin sheath as compared to the control cultures. Magnification: left panel = 10X, right panel = 40X. **(b)** Quantitative evaluation: FG1 promotes myelination ****P <0.0001. Error bars represent SEM of two experiments. Myelinating cultures were treated with 100 ng/ml FGF1 for different time periods. Axonal density calculated as pixel for NFL (different FGF1 dosages/control) were 1.17 for 5 ng/ml FGF1, 1.2 for 50 ng/ml FGF1 and 1.06 for 50 ng/ml FGF1. **(c)** 16 days (12 DIV to 28 DIV) and **(d)** 6 days (12 DIV to 18 DIV). The myelination was enhanced at day 18 and 24, but unaltered at day 28. ****P <0.0001. Significance of data values was analyzed using T-test. Error bars represent SEM from three independent experiments. The axonal densities (FGF1/control) ranged between 0.98 and 1.02 in the experiments shown in **(c)** and **(d)**.

Three independent conditions were used to assess ability of FGF1 to stimulate myelination in these cultures. First, FGF1 was added for four days after the onset of myelination and this enhanced myelination up to 1.7-fold (p < 0.0001) (Figure 5a and 5b). Second, FGF1 was added before the onset of myelination at day 12 for a total of 16 days. Again, we observed FGF1 enhanced myelination, particularly at 18 and 24 days in vitro (DIV) (Figure 5c). Third, FGF1 was added before the onset of myelination for 6 days (12–18 DIV) and then withdrawn while myelination was still ongoing. Enhanced myelination could already be detected during the initial phase of myelination, was still seen six days after FGF1 was withdrawn and then disappeared thereafter (Figure 5d). This indicates that FGF1 accelerates myelination in this culture system.

### FGF1 promotes remyelination in cerebellar slice cultures

**Figure 6:**
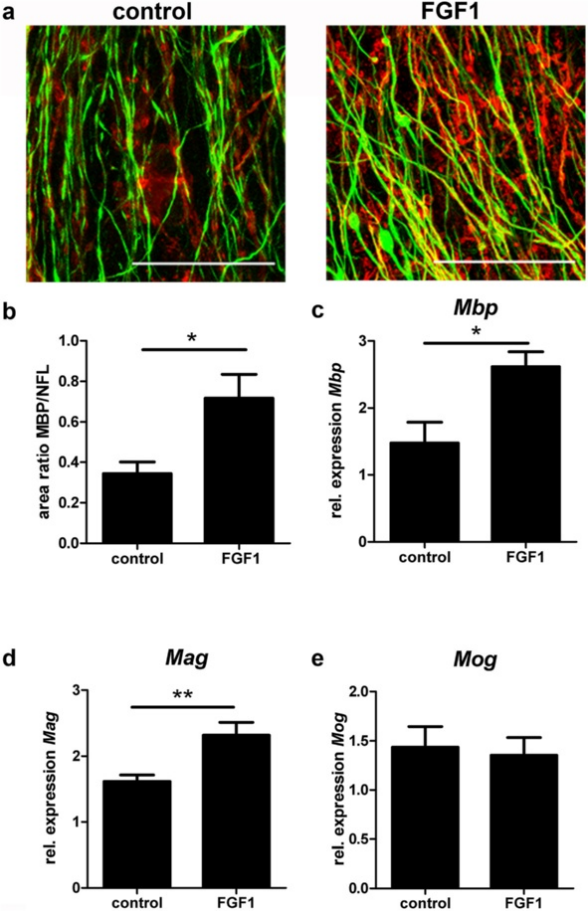
**FGF1 enhances remyelination in organotypic cerebellar slice cultures.** After toxic demyelination, cerebellar slice cultures were allowed to remyelinate for 14 days in the absence or presence of 100 ng/ml FGF1. Myelination was assessed by immunostaining **(a, b)** and quantitative PCR **(c–e)**. **(a)** *Mpb* (red), NFL (green) scale bar = 50 μm **(b)** Quantification of *Mpb* ^+^/NFL^+^ area ratio in FGF1 treated slices compared to untreated controls. *Mpb* ^+^ myelin formation is promoted by FGF1. Students t-test *P = 0.0341. **(c-e)** After 14 days RNA was extracted, cDNA obtained and transcript levels of **(c)** *Mpb*, **(d)** *Mag* and **(e)** *Mog* were measured by qPCR. One-way ANOVA **P < 0.01; *P < 0.05; All error bars represent SEM from three independent experiments.

To determine whether FGF1 would also promote remyelination, we investigated its effects on demyelinated organotypic cerebellar cultures derived from newborn mouse pups. Slice cultures myelinated *in vitro* in 12 days, they were then demyelinated using lysolecithin and allowed to recover in the presence or absence of FGF1. Analysis of cultures 14 days later revealed that FGF1 promoted remyelination; myelin basic protein (MBP) immunoreactivity increased approximately 1.5-fold as assessed by immunofluorescence microscopy (Figure 6a and 6b). The MBP expression was normalized based on the axonal density as measured by NFL staining. This increase in MBP positive immunoreactivity was paralleled by an increase of *Mbp* transcripts (Figure 6c). Further, we observed an increase in transcript level of *Mag* (Figure 6d), but not consistently of *Mog* (Figure 6e).

### FGF1 decelerates differentiation of monocultured OPCs

**Figure 7:**
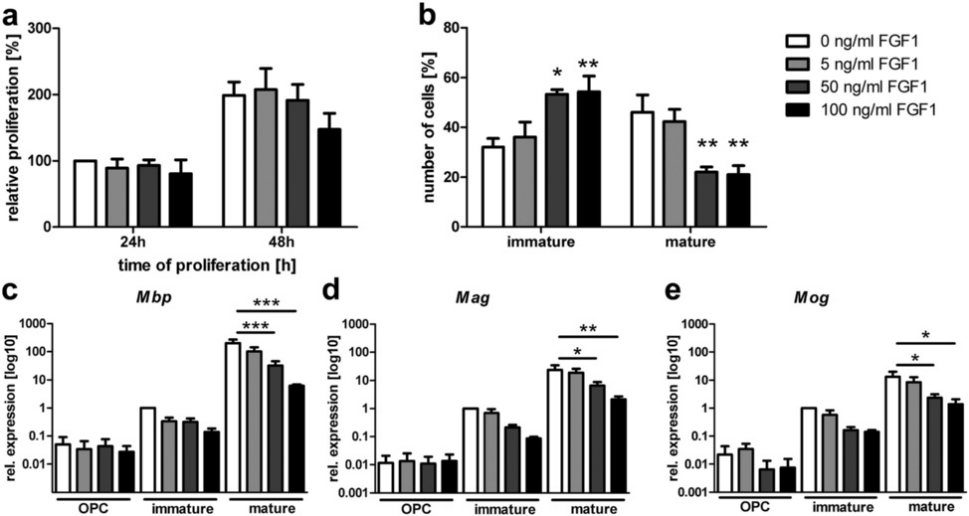
**FGF1 decelerates** **differentiation of monocultured oligodendrocytes. (a)** FGF1 does not affect proliferation of primary murine OPCs but **(b)** decelerates the differentiation into myelinating oligodendrocytes as determined by morphology; Two-way ANOVA with Bonferroni post correction **p < 0.01; *p < 0.05. The expression of myelin-associated genes was assessed by qPCR on samples from OPCs (6 h), immature (24 h) and mature cells (48 h). The expression levels of **(c)** *Mbp*, **(d)** *Mag* and in **(e)** *Mog* are reduced due to increasing FGF1 concentrations; one-way ANOVA with Bonferroni post correction *** p < 0.001 **P < 0.01; *P = <0.05; error bars represent SEM from three independent experiments.

To determine the effect of FGF1 on the proliferation of monocultured oligodendroglial cells we cultured OPCs in the presence of PDGF-AA, NT3 and different concentrations of FGF1 for 24 or 48 h. No significant influence on the proliferation capacity of OPCs after 24 and 48 h compared to controls could be shown by BrdU assays (Figure 7a). To analyse effects of FGF1 on oligodendroglial differentiation, different concentrations of FGF1 were added to differentiating oligodendrocytes for up to 48 h. The differentiation of oligodendrocytes was analyzed using morphological parameters as well as the mRNA expression levels of several genes coding for myelin proteins. Addition of FGF1 for 48 h decreased significantly the number of mature oligodendrocytes characterized by multiple processes and complex branching (Figure 7b). To further examine the effect on oligodendroglial differentiation, we analyzed the mRNA expression levels of myelin proteins *(Mbp, Mag,* and *Mog)* at different time points. The expression levels of all genes were significantly downregulated after exposure to FGF1 (Figure 7c-e).

### FGF1 induces transcriptional changes in human astrocytes including upregulation of *CXCL8* and *LIF*

**Figure 8:**
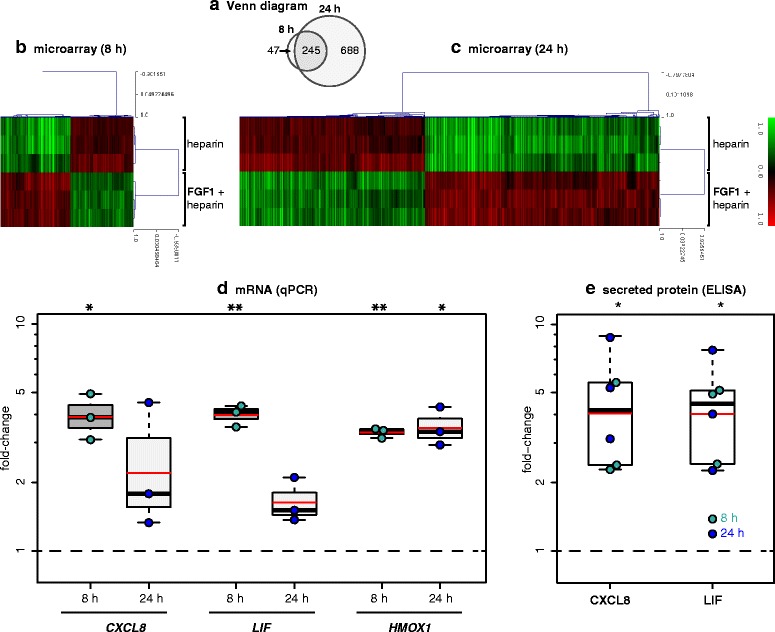
**FGF1-induced gene expression in human primary astrocytes. (a-c)** Gene expression was analysed after 8 h **(a,b)** and 24 h **(a,c)** with and without FGF1 (10 ng/ml). In all experiments, FGF1 was applied together with heparin (5 U/ml); control cultures contained heparin only. Only differentially expressed probes (corrected p-value <0.05, fold-change > 1.4-fold up or down, and normalized mean expression intensity ≥ 100 in any one of the two groups) are shown. The number of differentially expressed probes is shown in **(a)**. *CXCL8* and *LIF* were among the genes upregulated at both time points (see also Additional file 5: Table S3). **(d)** For validation, the gene expression of *CXCL8*, *LIF*, and the positive control *HMOX1* were analysed by qPCR after 8 h and 24 h with FGF1 (10 ng/ml). **(e)** Secreted *CXCL8* and *LIF* protein were measured by ELISA in the supernatant after 8 and 24 h with and without 10 ng/ml FGF1. In **(d,e)**, the fold-change compared to unstimulated control cultures is displayed. Black bars and boxes indicate medians and 1^st^/3^rd^ quartiles, respectively. Whiskers extend to the most extreme samples up to 1.5× of the IQR. Red bars indicate means. Fold-changes were analysed by two-sided one-sample tests against the control samples (μ = 1; t-test for FGF1 mRNA, U test for FGF1 protein because of non-normal distribution). *: p < 0.05, **: p < 0.01.

Since *FGF1* is highly abundant in MS lesions (Figure 1) and astrocytes are known to express FGFRs [55,56], we analyzed transcriptional responses of human astrocytes to FGF1. First, we established the response of our cultured human astrocytes to FGF1 by analyzing the induction of *HMOX1*, which has been reported to be induced in rodent astrocytes by FGF1 [56]. The interaction of FGF1 with its receptors is stabilized and stimulation efficacy is increased by binding to heparan sulfate [57-60]. Experiments to establish appropriate stimulation conditions showed that 5 U/ml heparin and 10 ng/ml FGF1 caused a reliable up-regulation of *HMOX1*, while heparin alone had no effect (Figure 8 and data not shown).

Further, the effects of FGF1 stimulation on primary human astrocytes were investigated using Affymetrix GeneChip® Human Gene 1.0 ST Array. Triplicate samples were analyzed at 8 and 24 h. Differential expression analysis between FGF-treated astrocytes and control cultures identified 292 probe sets (154 upregulated and 138 downregulated) at 8 h and 933 probe sets (521 upregulated and 412 downregulated) at 24 h (Figure 8a-c). Accordingly, unsupervised hierarchical clustering based on the transcriptional signatures at 8 and 24 h clustered together the FGF1 replicates and classified them apart from the control replicates (heatmaps in Figure 8b, c). Interestingly, 245 out of 292 (about 85%) of differentially expressed probe sets at 8 h maintained differential expression at 24 h. All probe sets regulated by FGF1 at 8 and 24 h and the genes they represent are shown in Additional file 5: Table S3. Among these genes regulated by FGF1 were two released factors that had been linked previously to remyelination, namely *LIF* and *CXCL8*. We then validated the induction of *CXCL8* and *LIF* in human astrocytes by FGF1 further by both qPCR and ELISA (Figure 8d, e).

## Discussion

This study presents a quantitative expression analysis of remyelinated MS lesions. We describe a complex change of multiple factors known to affect oligodendrocyte proliferation and maturation. Among all 46 analyzed factors, *FGF1* was the most abundant one in remyelinated lesions and was further induced in these lesions. In functional experiments with dissociated myelinating cultures and remyelinating slice cultures, we found that FGF1 promotes both developmental myelination and remyelination.

FGF family members are widely expressed in the brain, and are recognized as determinants of neuronal survival during development and adulthood [61]. Here we identified a role for FGF1 in promoting remyelination with potential relevance for repair in human MS. This is consistent with previous findings showing a role of the FGF family in myelination [39,62,63].

In MS lesions, FGF1 was localized on astrocytes, neurons, oligodendrocytes, microglia and infiltrating T cells and B cells. Previously FGF1 was reported in neurons [64] and reactive astrocytes in Alzheimer’s disease [65]. In an animal model of chemically induced demyelination, enhanced expression of FGF1 was described during remyelination [66]. Our qPCR analysis indicated a higher level of *FGF1* also in active lesions. In addition, our immunostaining localized FGF1 on T cells and microglia in active MS lesions. FGF1 could be produced by these cells themselves or alternatively been picked up after release from surrounding cells. Our qPCR shows that in normal brain and in the different MS lesions, *FGF1* is abundantly transcribed, but the precise stimuli triggering FGF1 expression during experimental de- and remyelination and in remyelinated MS lesions remain to be identified.

MS lesions frequently show a demyelinated core and a remyelinating rim [11]. Examining two such tissue blocks we found *FGF1* to be higher in the remyelinated rim compared to the demyelinated core. This further indicates that success of remyelination is associated with increased levels of FGF1. The up-regulation of *FGF1* was found in remyelinated areas, in which the repair had already been carried out before. Therefore our tissue analysis would not allow concluding whether FGF1 is involved in active remyelination and/or maintenance of remyelinated myelin sheaths. Our functional experiments, however, indicate that FGF1 has a role in the active myelination and remyelination process. In an *in vitro* culture system recapitulating essential features of myelination [42-44] we found FGF1 to promote developmental myelination. The significance of the FGF family in developmental myelination has been established *in vivo*: Fibroblast growth factor receptor (FGFR) signaling is required for the generation of OPCs from the embryonic forebrain [63]. Remyelination and developmental myelination share essential features, but possible differences between development and repair have to be considered [67]. Therefore, we employed a slice culture model [45,48] and found that FGF1 promoted also remyelination.

Our further experiments with pure cultures of oligodendrocytes and astrocytes suggest that the (re)myelination promoting effect of FGF1 is mediated by an indirect mechanism: FGF1 promoted (re)myelination in mixed cultures and slice cultures that contained oligodendrocytes, microglia, and astrocytes. In pure oligodendrocyte cultures, however, FGF1 did not induce proliferation and inhibited the differentiation. Therefore we analyzed if FGF1 might induce myelination-promoting factors in astrocytes. We switched for these experiments to human astrocytes, since understanding of regulation of remyelination in MS is our purpose. We found that FGF1 induced LIF and CXCL8 in primary human astrocytes. CXCL8, which does not have a homologue in rats and mice, is not only a chemoattractant for monocytes and neutrophils, but also recruits oligodendrocyte progenitor cells [68]. CXCL8 was detected in astrocytes around active MS lesions [69]; its receptors CXCR2 and CXCR1 [70] were found on oligodendrocyte precursor cells [71] and, importantly, upregulated on oligodendrocytes around MS lesions [68]. CXCL8 was therefore suggested to be involved in regeneration of MS lesions [69]. LIF has been established to support oligodendrocyte survival *in vitro* [72,73] and *in vivo* [74,75] as well as to promote myelination [76]. In EAE models, LIF directly prevented oligodendrocyte cell death [74] and promoted remyelination [77]. Together, we found that human astrocytes produce CXCL8 and LIF in response to FGF1, and these findings together with our results obtained with rodent culture systems (myelinating dissociated cultures, remyelinating slice cultures, and pure oligodendrocytes) indicate that FGF1 promotes the induction of (re)myelination indirectly via an effect mediated by astrocytes, although the relative importance of different FGF1-induced factors in the rodent models of remyelination remains to be specified. One constant feature of remyelination is that the repaired myelin is thinner than the original myelin [78], but it is unclear why this is the case. Previous findings that FGFR signaling regulates myelin thickness in development [39] raise the possibility that FGFs may participate in the regulation of this aspect of remyelination.

Beyond the FGF-family, our study provides quantitative information on additional regulators of oligodendrocytes biology in de- and remyelinated human MS lesions such as Lingo-1, which is a target in current clinical trials [27,54]. Semaphorins were originally identified as axonal guidance cues, but they also regulate migration and oligodendrocyte biology [29,31,79]. We noted that 5 semaphorins (*3C, 4D, 6A, 6D,* and *7A*) were significantly higher expressed in re- versus demyelinated lesions. *Sema6A* and *Sema6D* had not been linked before to remyelination in MS. All this inspires further studies on the role of semaphorins in the regulation of remyelination in MS.

Mediators commonly used to promote oligodendrocyte cultures such as FGF2, PDGF, ciliary neurotrophic factor (CNTF), IGFs, and IL-11 were present or even enhanced in inactive demyelinated MS lesions. This adds to the view that it is not the scarcity or even lack of one specific factor in the inactive demyelinated lesions that can explain the failure of remyelination. Since myelination depends on a delicate and fine-tuned balance of many factors [78], the multiple alterations observed here (Table 1) may well contribute to the failure of remyelination together with the induction of inhibitory mechanisms.

In summary, we present a quantitative analysis of oligodendrocyte regulators in remyelinated MS lesions. We report that success of remyelination in MS is accompanied by high levels of *FGF1* and that FGF1 enhances myelination and remyelination *in vitro* presumably via an indirect mechanism mediated by astrocytes. Targeting FGF family members may have therapeutic potential [80], and should be tested to enhance repair in MS.

## Additional files

Additional file 1: Table S1. Frozen tissue samples used for qPCR and immunofluorescence. Additional file 2: Figure S1. Unsupervised clustering reflects histological discrimination between remyelinated (blue) and demyelinated inactive (pink) lesion areas. Additional file 3: Table S2. Expression levels of receptors and their fold changes in demyelinated inactive versus remyelinated lesions. Additional file 4: Figure S2. LRRN6A (LINGO-1) gene expression in different MS lesion types and control white matter. Additional file 5: Table S3. Transcriptional changes induced by FGF1 in human primary astrocytes, after 8 and 24 h.

## Abbreviations

ABI: Applied Biosystems
BAFF: B cell activating factor of the TNF family
CNTF: Ciliary neurotrophic factor
DAB: Diaminobenzidine
DIV: Day *in vitro*
FFPE: Formalin fixed paraffin embeded
FGF: Fibroblast growth factor
FGFR: Fibroblast growth factor receptor
GFAP: Glial fibrillary acidic protein
hARP: Human acid ribosomal protein
IGF: Insulin derived growth factor
LDA: Low density array
LIF: Leukemia inhibitory factor
LFB: Luxol fast blue
MAG: Myelin associated glycoprotein
MBP: Myelin basic protein
MOG: Myelin oligodendrocyte glycoprotein
MS: Multiple sclerosis
OPCs: Oligodendrocyte progenitor cells
P0: Newborn
PAP: Peroxidase anti peroxidase
PDGF: Platelet derived growth factor
PPIA: Peptidylprolyl isomerase A
qPCR: Quantitative PCR
RT: Room temperature
