# Supplementary material for: Transcript profiling of different types of multiple sclerosis lesions yields FGF1 as a promoter of remyelination
Source: Acta Neuropathol Commun. 2014 Dec 11;2:168. doi: 10.1186/s40478-014-0168-9 (PMC4359505; doi:10.1186/s40478-014-0168-9)
Supplement: Additional file 1: Table S1. — Frozen tissue samples used for qPCR and immunofluorescence. [file 40478_2014_168_MOESM1_ESM.pdf]

Supplementary Table 1: Frozen tissue samples used for qPCR and immunofluorescence

patients and controls	blocks	lesion type and control	age (Y)	sex	disease duration (Y)	MS classification
MS1	1	active	52	F	11	SPMS
MS2	2	inactive and remyelinated	50	F	17	SPMS
MS3	1	active	48	F	8	SPMS
MS4	1	inactive	65	M	25	SPMS
MS5	2	active	53	F	16	SPMS
MS6	3	remyelinated	59	M	32	SPMS
MS7	1	active	53	F	22	SPMS
MS8	2	active and remyelinated	66	M	--	--
MS9	1	remyelinated	70	F	40	PPMS
MS10	3	inactive and remyelinated	77	F	50	RRMS
MS11	1	inactive	42	M	6	PPMS
MS12	1	remyelinated	81	F	41	SPMS
MS13	1	remyelinated	47	F	24	SPMS
MS14	1	remyelinated	54	F	--	--
MS15	1	remyelinated	62	M	--	--
C1	1	control	47	F		
C2	3	control	46	F		
C3	3	control	49	M		
C4	3	control	48	M		
C5	1	control	18	M		
C6	1	control	16	M		
C7	1	control	66	F		

F: female; M: male; PPMS: primary progressive multiple sclerosis; RRMS: relapsing remitting multiple sclerosis; SPMS: secondary progressive multiple sclerosis; Y: years; -- disease duration and MS classification not exactly known.
