# Supplementary material for: Transcript profiling of different types of multiple sclerosis lesions yields FGF1 as a promoter of remyelination
Source: Acta Neuropathol Commun. 2014 Dec 11;2:168. doi: 10.1186/s40478-014-0168-9 (PMC4359505; doi:10.1186/s40478-014-0168-9)
Supplement: Additional file 2: Figure S1. — Unsupervised clustering reflects histological discrimination between remyelinated (blue) and demyelinated inactive (pink) lesion areas. [file 40478_2014_168_MOESM2_ESM.pdf]

Supplementary Figure 1

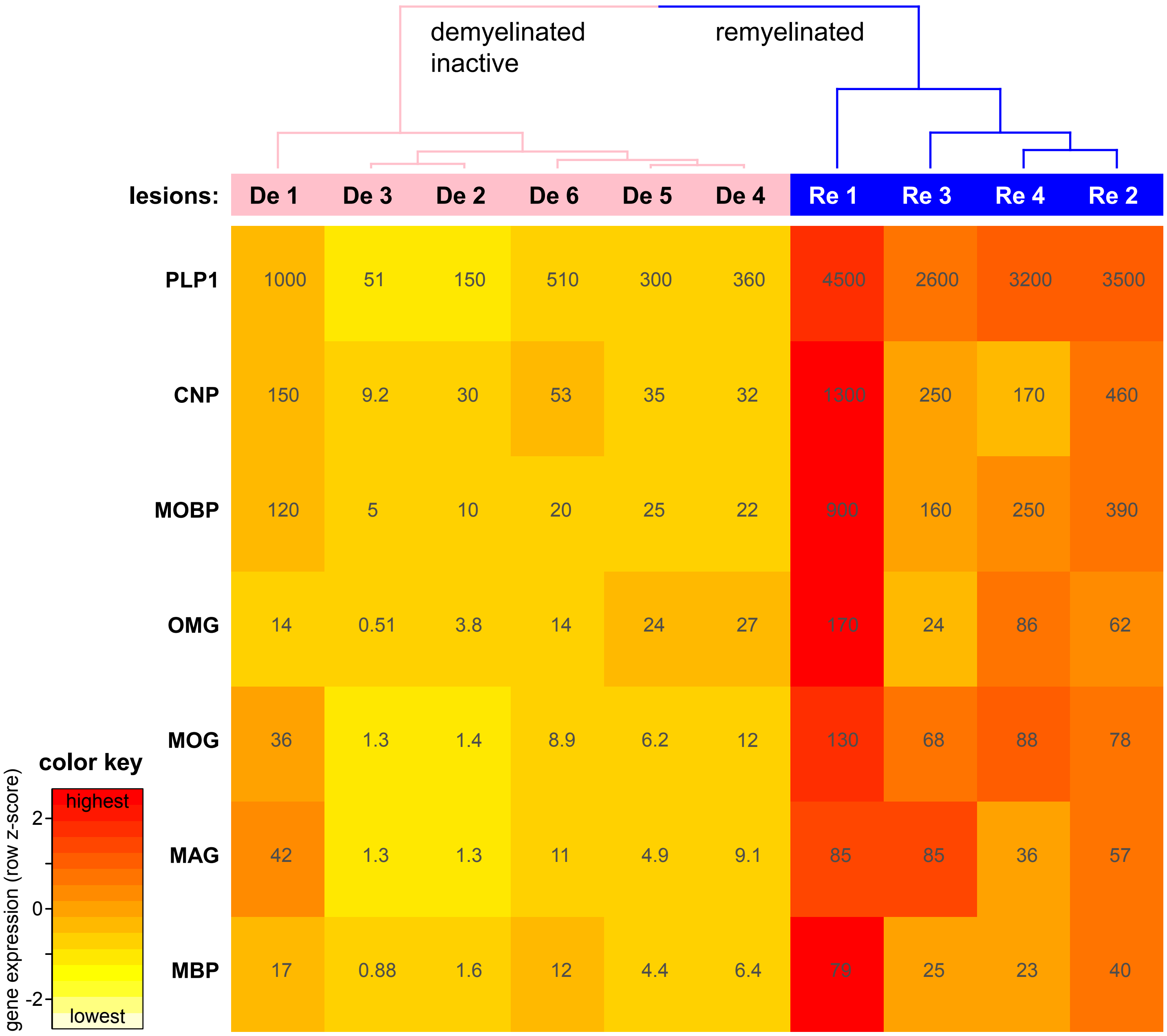

Suppl. Figure 1: Unsupervised clustering reflects histological discrimination between remyelinated (blue) and demyelinated inactive (pink) lesion areas. Gene expression of myelin proteins was analyzed by quantitative PCR on low density TaqMan arrays. These values were normalized to the housekeeping gene *GAPDH* (values in % *GAPDH* displayed within heatmap) and then color-coded as z-scores along rows (i.e. separately for each gene, all sample values were centered to mean=0 and scaled to SD=1; the yellows are the samples with the lowest and the reds the samples with the highest gene expression), in order to visualize expression of genes with high *(PLP1)* and low *(MAG, MOG, MBP, OMG)* expression within the same figure. Unsupervised hierarchical clustering was performed using R [1] version 3.0.1, function `heatmap.2` in package `gplots` [2], with clustering function `hclust(d, method="complete")` and distance matrix calculation by `dist(x, method="euclidean")`. Re: remyelinated lesions (blue); De: demyelinated inactive lesions (pink).

Supplementary references

1. R Core Team (2013). R: A language and environment for statistical computing. R Foundation for Statistical Computing, Vienna, Austria. URL: http://www.R-project.org/.

2. Gregory R. Warnes, Ben Bolker, Lodewijk Bonebakker, Robert Gentleman, Wolfgang Huber Andy Liaw, Thomas Lumley, Martin Maechler, Arni Magnusson, Steffen Moeller, Marc Schwartz and Bill Venables (2014). gplots: Various R programming tools for plotting data. R package version 2.14.1. URL: http://CRAN.R-project.org/package=gplots
