# Supplementary material for: Transcript profiling of different types of multiple sclerosis lesions yields FGF1 as a promoter of remyelination
Source: Acta Neuropathol Commun. 2014 Dec 11;2:168. doi: 10.1186/s40478-014-0168-9 (PMC4359505; doi:10.1186/s40478-014-0168-9)
Supplement: Additional file 3: Table S2. — Expression levels of receptors and their fold changes in demyelinated inactive versus remyelinated lesions. [file 40478_2014_168_MOESM3_ESM.docx]

**Supplementary Table 2: Expression levels of receptors and their fold changes in demyelinated inactive versus remyelinated lesions.**

| **Gene name** | **CWM** | | **Remyelinated** | | **Demyelinated** | | **Active** | | *Re/‌CWM* |  | *Re/De* |  |
| --- | --- | --- | --- | --- | --- | --- | --- | --- | --- | --- | --- | --- |
|  | mean | SEM | mean | SEM | mean | SEM | mean | SEM | *ratio* | p | *ratio* | p |
| **EGFRs** |  |  |  |  |  |  |  |  |  |  |  |  |
| *EGFR* | **1.67** | 0.34 | **3.44** | 0.66 | **6.75** | 2.44 | **6.78** | 5.73 | *2.06* | 0.067 | *0.51* | 1.00 |
| *ERBB3* | **41.06** | 13.07 | **73.67** | 30.23 | **9.63** | 3.83 | **43.62** | 28.08 | *1.79* | 0.48 | *7.65* | **0.010** |
| *ERBB4* | **3.41** | 0.96 | **5.24** | 1.29 | **4.42** | 0.84 | **6.26** | 3.35 | *1.53* | 0.35 | *1.19* | 0.91 |
| **FGFRs** |  |  |  |  |  |  |  |  |  |  |  |  |
| *FGFR1* | **2.97** | 0.26 | **5.51** | 0.17 | **10.78** | 3.91 | **18.25** | 12.70 | *1.86* | **0.010** | *0.51* | 0.48 |
| *FGFR2* | **12.19** | 1.51 | **18.44** | 0.77 | **8.91** | 2.53 | **23.34** | 9.15 | *1.51* | **0.038** | *2.07* | **0.038** |
| *FGFR3* | **4.72** | 2.01 | **11.98** | 1.45 | **18.47** | 6.93 | **11.71** | 10.62 | *2.54* | 0.067 | *0.65* | 1.00 |
| *FGFR4* | **0.05** | 0.01 | **0.12** | 0.03 | **0.36** | 0.15 | **1.00** | 0.94 | *2.44* | 0.11 | *0.34* | 0.61 |
| **PDGFRs** |  |  |  |  |  |  |  |  |  |  |  |  |
| *PDGFRA* | **5.03** | 0.60 | **10.03** | 2.47 | **3.68** | 0.75 | **3.22** | 0.07 | *2.00* | 0.17 | *2.72* | 0.11 |
| *PDGFRB* | **1.52** | 0.19 | **3.56** | 0.47 | **8.01** | 2.85 | **7.36** | 5.78 | *2.33* | **0.010** | *0.44* | 1.00 |
| **IGFRs** |  |  |  |  |  |  |  |  |  |  |  |  |
| *IGF1R* | **4.55** | 1.29 | **10.41** | 3.31 | **10.56** | 3.86 | **46.64** | 41.32 | 2.29 | 0.067 | 0.99 | 0.91 |
| *IGF2R* | **0.95** | 0.13 | **1.64** | 0.25 | **1.36** | 0.44 | **4.64** | 3.22 | 1.72 | 0.067 | 1.20 | 0.35 |
| **IL6 family receptors** |  |  |  |  |  |  |  |  |  |  |  |  |
| *IL6ST* | **28.60** | 4.22 | **56.63** | 5.59 | **39.66** | 14.58 | **30.26** | 2.61 | *1.98* | **0.010** | *1.43* | 0.17 |
| *LIFR* | **0.00** | 0.00 | **0.00** | 0.00 | **0.03** | 0.03 | **0.00** | 0.00 | *0.00* | 0.071 | *0.00* | 0.54 |
| *CNTFR* | **3.04** | 0.44 | **2.66** | 0.69 | **8.09** | 3.16 | **15.55** | 13.52 | *0.87* | 0.76 | *0.33* | 0.76 |
| **Other receptors** |  |  |  |  |  |  |  |  |  |  |  |  |
| *MET* | **0.55** | 0.18 | **0.62** | 0.19 | **0.14** | 0.07 | **0.50** | 0.16 | *1.13* | 0.91 | *4.55* | **0.042** |
| *PLXNB1* | **5.98** | 1.43 | **17.11** | 4.22 | **9.05** | 3.67 | **20.60** | 16.46 | *2.86* | **0.019** | *1.89* | 0.26 |
| *IL11RA* | **3.31** | 0.50 | **2.82** | 0.42 | **5.01** | 1.74 | **4.58** | 2.21 | *0.85* | 0.48 | *0.56* | 1.00 |
| *CXCR1* | **0.01** | 0.01 | **0.01** | 0.01 | **0.02** | 0.01 | **0.00** | 0.00 | *1.11* | 0.67 | *0.77* | 1.00 |
| *CXCR2* | **0.05** | 0.02 | **0.01** | 0.01 | **0.06** | 0.04 | **0.66** | 0.60 | *0.12* | 0.067 | *0.10* | 0.34 |
| *CXCR3* | **0.00** | 0.00 | **0.00** | 0.00 | **0.01** | 0.01 | **0.02** | 0.01 | *1.04* | 0.90 | *0.12** | 1.00 |
| *CXCR4* | **0.58** | 0.06 | **0.93** | 0.23 | **0.99** | 0.43 | **5.07** | 3.40 | *1.60* | 0.26 | *0.94* | 0.91 |
| *CXCR7* | **0.55** | 0.03 | **1.46** | 0.11 | **2.79** | 1.16 | **1.28** | 0.49 | *2.67* | **0.010** | *0.52* | 0.48 |

CWM: control white matter, Remyelinated: remyelinated lesion, Demyelinated: demyelinated inactive lesion, Active: demyelinated active lesion, ratio De/‌Re: demyelinated inactive versus remyelinated lesions. 0.00: Values were below our detection limit, which was 0.01% *GAPDH.* 6 normal white matter specimens from 4 subjects, 6 demyelinated inactive lesions from 4 subjects, 4 demyelinated active lesions from 3 subjects and 4 remyelinated lesions from 3 subjects were dissected and used for qPCR analysis. The mean expression values are given as % *GAPDH*; SEM denotes standard error of the mean. ***** Ratio not meaningful, because both values were close to zero**.**
