# Supplementary material for: Transcript profiling of different types of multiple sclerosis lesions yields FGF1 as a promoter of remyelination
Source: Acta Neuropathol Commun. 2014 Dec 11;2:168. doi: 10.1186/s40478-014-0168-9 (PMC4359505; doi:10.1186/s40478-014-0168-9)
Supplement: Additional file 4: Figure S2. — LRRN6A (LINGO-1) gene expression in different MS lesion types and control white matter. [file 40478_2014_168_MOESM4_ESM.pdf]

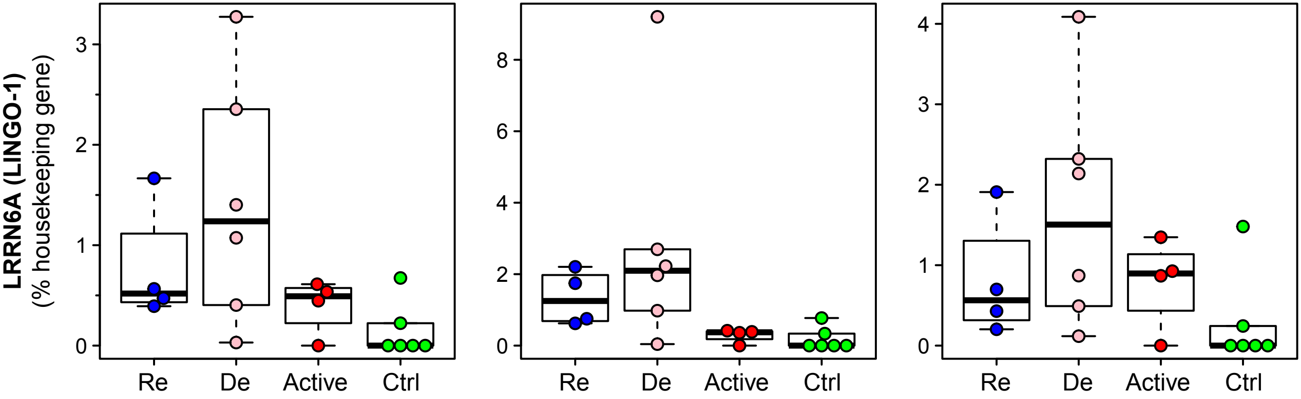

housekeeping gene: **GAPDH** **ACTB** **PPIA**

Suppl. Figure 2: LRRN6A (LINGO-1) gene expression in different MS lesion types and control white matter. LINGO-1 was quantified by qPCR TLDA and is shown normalized to the 3 housekeeping genes GAPDH, ACTB, and PPIA. Each symbol represents one dissected lesion area or control tissue. Re: remyelinated, De: demyelinated inactive, Active: actively demyelinating lesions; Ctrl: control white matter. Boxplots include medians, 1st/3rd quartiles, and whiskers extending up to 1.5x of the interquartile range.
